# The Impact of GLP-1 Receptor Agonists (GLP-1 RAs) on Mental Health: A Systematic Review

**DOI:** 10.1192/j.eurpsy.2025.1993

**Published:** 2025-08-26

**Authors:** S. Tempia Valenta, A. Nicastri, F. Perazza, F. Marcolini, V. Beghelli, A. R. Atti, M. L. Petroni

**Affiliations:** 1 Department of Biomedical and Neuromotor Sciences; 2 Department of Medical and Surgical Sciences, Alma Mater University of Bologna, Bologna, Italy

## Abstract

**Introduction:**

Glucagon-like peptide-1 receptor agonists (GLP-1 RAs) are a class of pharmacological agents that have gained significant attention and utility in managing metabolic disorders, particularly type 2 diabetes mellitus and obesity, often in combination with behavioral therapy. These agents mimic the physiological actions of GLP-1, a hormone released from the gastrointestinal tract in response to food intake, thereby stimulating insulin secretion, suppressing glucagon release, slowing gastric emptying, and promoting satiety through both peripheral and central nervous system mechanisms. Beyond their metabolic benefits, GLP-1 RAs have shown potential antidepressant and anxiolytic effects, which may be mediated through neuroprotective mechanisms, reduction of neuroinflammation, and modulation of neurotransmitter systems.

**Objectives:**

This systematic review aims to synthesize existing evidence on the effects of GLP-1 RAs on mental health outcomes, including both potential therapeutic benefits across various psychiatric disorders and the risk of associated adverse mental health effects.

**Methods:**

This systematic review was conducted across PubMed/MEDLINE and Web of Science. Inclusion criteria encompassed human and animal studies examining the impact of GLP-1 RAs on mental health. Data from 81 selected studies were extracted and analyzed, focusing on mental health outcomes and reported adverse effects.

**Results:**

GLP-1 RAs exhibit potential beneficial effects on depressive symptoms, cognitive function, and reduced risk of suicidal ideation in animal and human models through antioxidative, anti-inflammatory mechanisms, and modulation of neurotransmitter pathways. Additionally, GLP-1 RAs were effective in reducing alcohol and substance use and binge eating behaviors. Adverse psychiatric effects associated with GLP-1 RAs, including depression, anxiety, and suicidal ideation, are noted in pharmacovigilance analyses, with variations among different GLP-1 RAs.

**Conclusions:**

Our systematic review highlights the potential role of GLP-1 RAs in psychiatric care, underscoring their potential therapeutic benefits across a spectrum of mental health conditions. Animal studies consistently demonstrate positive outcomes, revealing antidepressant effects, modulation of addictive behaviors, and improvements in eating disorders through various neurobiological mechanisms. Human studies present a more nuanced landscape with conflicting evidence regarding the impact of GLP-1 RAs on depressive symptoms and other psychiatric outcomes, suggesting a need for further rigorous investigation. Despite these promising findings, significant limitations, such as study heterogeneity, funding biases, and the predominance of animal model data, temper definitive conclusions. Longitudinal and rigorous studies are warranted to elucidate the long-term effects and safety profile of GLP-1 RAs in psychiatric contexts.

**Disclosure of Interest:**

None Declared

